# A novel therapeutic approach using peripheral blood mononuclear cells preconditioned by oxygen-glucose deprivation

**DOI:** 10.1038/s41598-019-53418-5

**Published:** 2019-11-14

**Authors:** Masahiro Hatakeyama, Masato Kanazawa, Itaru Ninomiya, Kaoru Omae, Yasuko Kimura, Tetsuya Takahashi, Osamu Onodera, Masanori Fukushima, Takayoshi Shimohata

**Affiliations:** 1 Department of Neurology, Brain Research Institute, Niigata University, 1-757 Asahimachi-dori, Chuoku Niigata, 951-8585 Japan; 2 Translational Research Center for Medical Innovation, Foundation for Biomedical Research and Innovation at Kobe, 2-2 Minatojima-Minamimachi, Kobe, 650-0047 Japan; 3 Department of Neurology, Gifu University Graduate School of Medicine, 1-1 Yanagido, Gifu, 501-1194 Japan

**Keywords:** Stroke, Stroke

## Abstract

Cell therapies that invoke pleiotropic mechanisms may facilitate functional recovery in patients with stroke. Based on previous experiments using microglia preconditioned by oxygen-glucose deprivation, we hypothesized that the administration of peripheral blood mononuclear cells (PBMCs) preconditioned by oxygen-glucose deprivation (OGD-PBMCs) to be a therapeutic strategy for ischemic stroke. Here, OGD-PBMCs were identified to secrete remodelling factors, including the vascular endothelial growth factor and transforming growth factor-β *in vitro*, while intra-arterial administration of OGD-PBMCs at 7 days after focal cerebral ischemia prompted expression of such factors in the brain parenchyma at 28 days following focal cerebral ischemia *in vivo*. Furthermore, administration of OGD-PBMCs induced an increasing number of stage-specific embryonic antigen-3-positive cells both *in vitro* and *in vivo*. Finally, it was found to prompt angiogenesis and axonal outgrowth, and functional recovery after cerebral ischemia. In conclusion, the administration of OGD-PBMCs might be a novel therapeutic strategy against ischemic stroke.

## Introduction

Cell therapies using either bone marrow mononuclear cells^1^, or bone marrow-derived mesenchymal stem/stromal cells^2–4^ was proposed as a therapeutic approach targeting multiple cell types to enhance protection and recovery via pleiotropic mechanisms after ischemic stroke^5^. These days, clinical application towards further development of cell therapies against ischemic stroke are accelerated. Actually, phase III clinical trial using bone marrow-derived mesenchymal stem cells against ischemic stroke is conducted (#JMA-IIA00117)^2^. Japanese health ministry this year also gave conditional approval for the traumatic spinal injury (#JMA-IIA00154). In addition, phase I clinical trials using stage-specific embryonic antigen-3 (SSEA-3)-positive stem cells are conducting for ischemic stroke^6,7^, acute myocardial infarction (#UMIN000019514)^8^, and epidermolysis bullosa in Japan. However, previous sources of cell therapies reported some problems, such as the clinical concern associated with the technical safety of bone marrow-derived cell collection in patients who are undergoing either anticoagulant or antiplatelet therapy for the secondary prevention of stroke as well as the need for general anaesthesia to harvest the bone marrow. Thus, cell processing centres are essential to culture these cells. Therefore, obtaining these cells is challenging, whereas the clinical application of peripheral blood mononuclear cells (PBMCs) as the cell source is much easier. Our research group has previously described that primary microglia preconditioned by 18 h oxygen-glucose deprivation (OGD) (OGD-microglia) assumed a polar protective phenotype^9^. Similarly, the optimal (under 18 h) OGD condition was found to prompt microglia to (i) migrate into the periinfarct lesion, (ii) secrete remodelling factors (e.g., the vascular endothelial growth factor (VEGF) and the transforming growth factor-beta (TGF-β)) which prompted angiogenesis^10–12^ and axonal outgrowth^13,14^, and (iii) upregulate both the VEGF and TGF-β in resident native endothelial cells, astrocytes, pericytes, neurons, and microglia through paracrine actions^9^. Finally, administration of OGD-microglia was reported to induce angiogenesis and axonal outgrowth as well as functional recovery following cerebral ischemia^9^. These findings are consistent with the accumulating evidence suggesting that, in addition to neuroinflammation, infiltrating monocyte-derived macrophages play a major role also in the subsequent processes of regeneration in the ischemic brain^15,16^. Considering that microglia and monocytes/macrophages polarisation to their protective phenotype implies a novel therapeutic mechanism against stroke^17^, we considered PBMCs, which include monocyte-derived macrophages^16^, as potential candidates for cell therapies.

PBMCs might be a promising candidate to cell therapies for the stimulation of regeneration considering that the OGD condition might induce phenotypic changes in PBMCs. While PBMCs contain a population of monocytes/macrophages, mesenchymal progenitor cells and stem cells^18–22^, they secrete remodelling factors, such as the VEGF, basic fibroblast growth factor, and TGF-β after ischemia^20,23–25^. PMBCs are known to recruit into the brain parenchyma following cerebral ischemia in the subacute and chronic phases^26^. In addition, PBMCs can cross the BBB, particularly in the ischemic condition^15,20,26^. Moreover, although clinical trials of administration of autologous mesenchymal stem cells were shown to improve patients’ functional outcome after ischemic stroke^2,3^, the mechanisms mediating such a recovery are yet to be fully understood. To the contrary, a recent study demonstrated that cells positive for a human embryonic stem cell marker, i.e., the SSEA-3, are mobilised from the bone marrow into the peripheral blood after ischemic stroke^22^. Kuroda *et al*. reported a unique subset of mesenchymal stem cells positive for SSEA-3^27^, is able to self-renew and differentiate into cells representative of all three germ layers (i.e., endodermal, ectodermal, and mesodermal) from a single cell. Additionally, administration of stem cells positive for SSEA-3 at the delayed subacute phase of cerebral ischemia facilitated the neural reconstruction and improved functions^6,28^. Given that PBMCs under ischemic condition might contain SSEA-3-positive stem cells, we speculated that cell therapy using PBMCs preconditioned by optimal ischemic condition (OGD) (OGD-PBMCs) might be a simple and convenient therapeutic strategy for ischemic stroke.

In summary, based on previous experiments using OGD-microglia, we hypothesised that intra-arterially administered OGD-PBMCs might cross the BBB, secrete remodelling factors in the brain parenchyma, increase SSEA-3-positive cells, and exert pleiotropic therapeutic effects against focal cerebral ischemia through the promotion of angiogenesis and axonal outgrowth, even in the subacute phase. To test this hypothesis, we investigated whether the primary rat and human OGD-PBMCs polarised to the protective phenotype promoted functional recovery following focal cerebral ischemia in experimental ischemic stroke.

## Results

### The OGD condition is superior to both oxygen deprivation alone and glucose deprivation alone for VEGF secretion in PBMCs

The experiments were performed using human PBMCs to determine which condition amongst OGD, oxygen deprivation alone and glucose deprivation alone was superior to secrete VEGF. To isolate the PBMC fractions, Ficoll-Hypaque centrifugation was performed and the secretion of VEGF under the OGD condition was demonstrated to be significantly higher than that under the oxygen deprivation alone or normoxic condition. Thus, secretion of VEGF from human PBMCs under the OGD condition may be superior to the oxygen deprivation condition (Supplementary Figure 1). This indicated that the OGD condition may be suitable for the switch into the protective polarisation in PBMCs like microglia^9^.

### The 18-h OGD condition was superior to 6-h and 30-h OGD conditions for VEGF secretion in PBMCs

To evaluate the temporal effect of OGD on VEGF secretion by PBMCs, we measured the level of VEGF per cell in rat PBMC conditioned media after 6, 18, and 30 h incubation with OGD. Based on the enzyme-linked immunosorbent assay (ELISA) results, VEGF secretion was higher after 18-h OGD-PBMC treatment than after 6 h (Supplementary Figure 2), whereas 30-h OGD treatment resulted in cell death. Therefore, 18 h was the optimum treatment duration for this study.

### Marked changes in the levels of the VEGF and TGF-β secreted from OGD-PBMCs

**Figure 1:**
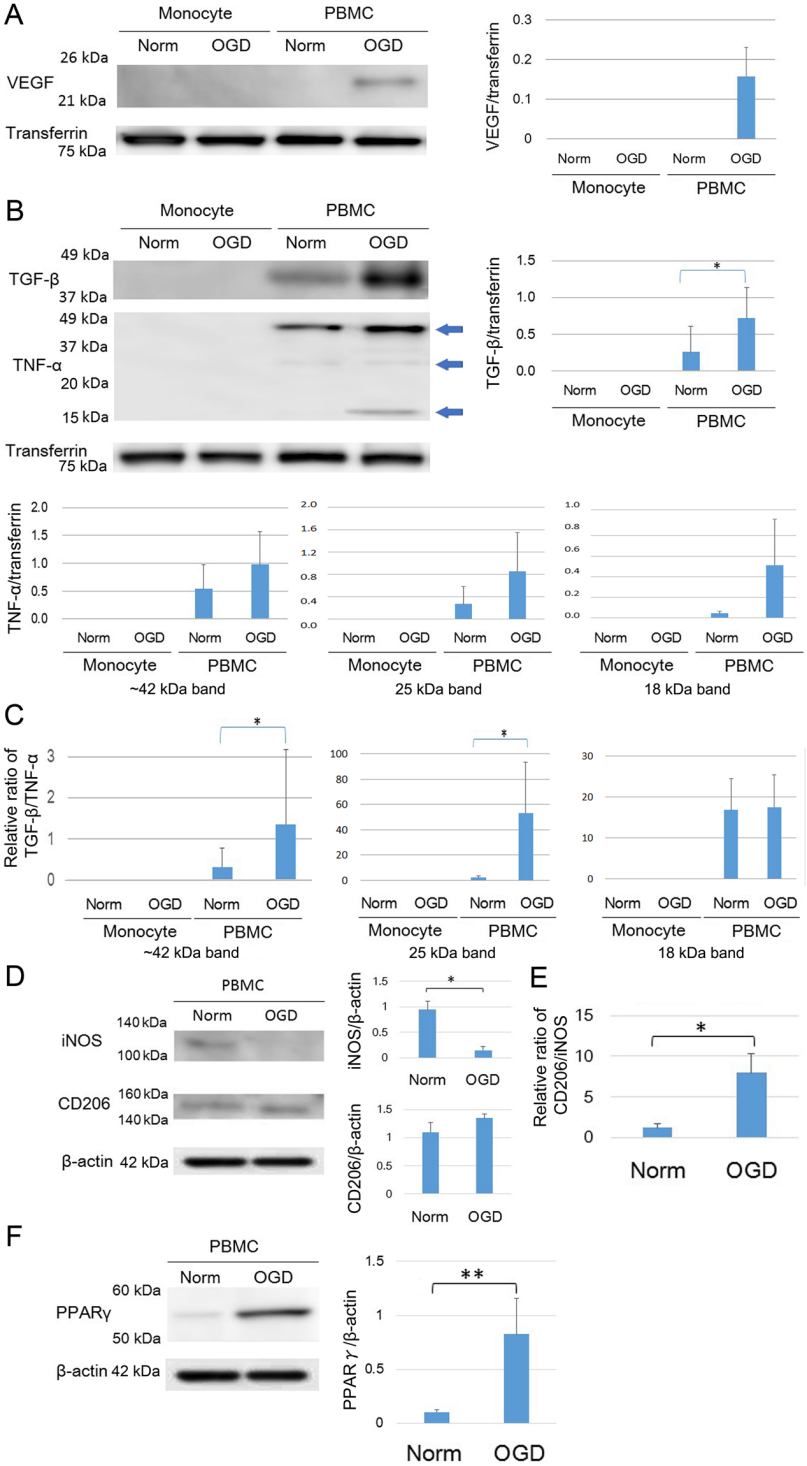
Characteristics of rat primary-cultured peripheral blood mononuclear cells (PBMCs) preconditioned by oxygen-glucose deprivation (OGD) (OGD-PBMCs). The levels of the secretory vascular endothelial growth factor (VEGF) (N = 4) **(A)**, anti-inflammatory cytokine transforming growth factor-β (TGF-β) and pro-inflammatory cytokine tumour necrosis factor-α (TNF-α) (N = 7 each) **(B)** from the conditioned media of rat primary-cultured PBMCs and isolated monocytes subjected to either normoxia (norm) or OGD. Western blotting for TNF-α showed a band around 42 kDa, a weak 25 kDa band and an 18 kDa band (arrow). (**C**) The ratio of TGF-β per TNF-α, which reflects the PBMCs polarisation, under both the normoxic and OGD conditions (N = 7 each). The increase in the ratio of TGF-β per TNF-α reveals the polarisation to the protective anti-inflammatory status under the OGD condition. (**D**) Representative figures indicate the expression of the pro-inflammatory marker (i.e., inducible nitric oxide synthase, iNOS) and the protective anti-inflammatory marker (cluster of differentiation 206, CD206) in the cell lysate from PBMCs under both the normoxic and the OGD conditions (N = 6 each). (**E**) The ratio of CD206 per iNOS, which reflects the PBMC polarisation under both the normoxic and OGD conditions (N = 6 each). The increase in the ratio of CD206 per iNOS reveals the polarisation of the protective anti-inflammatory status under the OGD condition. (**F**) Representative figures indicate the expression of the peroxisome proliferator-activated receptor γ (PPARγ) in the cell lysate from PBMCs under both the normoxic and the OGD conditions (N = 6 each). Transferrin and β−actin confirmed an equal loading of proteins. Data represent the relative optical densities of each sample compared to the loading control band by unpaired t-test. ^*^P < 0.05. ^**^P < 0.01.

To investigate the secretion of protective remodelling factors from OGD-PBMCs, the levels of the VEGF and TGF-β in the conditioned media of both PBMCs and isolated monocyte/macrophages under the normoxic and OGD conditions (18 h) were compared by western blotting. Briefly, while monocytes/macrophages were isolated by magnetic cell sorting (MACS) using an anti-CD11b antibody, their purity was >99%, as determined by the Mac-1 (CD11b/18) immunopositivity in the flow cytometry (Supplementary Figure 3). While VEGF levels were found in the conditioned media of OGD-PBMCs, the same was not valid for PBMCs under the normoxic condition (P = 0.029) (Fig. 1A). In addition, the level of TGF-β in the conditioned media of OGD-PBMCs were higher than that under the normoxic condition (P = 0.044). Western blotting for TNF-α showed a band around 42 kDa, a weak 25 kDa band and an 18 kDa band. However, differences of the level of TNF-α in the conditioned media between the OGD and normoxic conditions was not found (P = 0.19, 0.19, and 0.12, respectively) (Fig. 1B). Successively, the ratio of TGF-β to TNF-α was determined, as it reflects the polarisation of the protective anti-inflammatory and toxic pro-inflammatory monocytes/macrophages^29^ and microglia^9^. The ratio of TGF-β to TNF-α around 42 kDa and 25 kDa bands from OGD-PBMCs was higher than that from PBMCs under the normoxic condition (P = 0.044 and 0.027) (Fig. 1C). Finally, the levels of VEGF, TGF-β and TNF-α in the conditioned media from monocytes under both the normoxic and OGD conditions were not detected in these experiments (Fig. 1A,B; the long length western blots were also presented in Supplementary Figure 4A,B).

### Priming of the protective anti-inflammatory switch in the OGD-PBMCs

The polarisation of OGD-PBMCs on cell surface markers was investigated using antibodies against the inducible nitric oxide synthase (iNOS, i.e., a pro-inflammatory marker) and CD206 (i.e., a protective anti-inflammatory marker) (Fig. 1D). Western blotting revealed the level of iNOS in the OGD-PBMC cell lysates to be lower than that in the normoxic PBMC cell lysates (P = 0.01) (Fig. 1D; the long length western blots were also presented in Supplementary Figure 4C). In contrast, the same was not valid for the levels of CD206 (P = 0.94). The ratio of the levels of CD206 per iNOS, which reflects the polarisation of the protective anti-inflammatory and toxic pro-inflammatory monocytes/macrophages^29^ and microglia^9^, was higher in the OGD condition than the normoxic condition (P = 0.023) (Fig. 1E).

### Mechanism of PBMCs polarisation by OGD

To determine the mechanism by which optimal OGD influences the polarisation of PBMCs, we evaluated the level of transcription factor peroxisome proliferator-activated receptor-γ (PPARγ). PPARγ, a ligand-activated nuclear receptor, exerts potent anti-inflammatory properties through its upregulation and also enables protective differentiation of monocytes into macrophages^30^. Western blotting revealed the level of PPARγ to be higher in the OGD-PBMC cell lysates than the normoxic PBMC cell lysates (P < 0.001) (Fig. 1F; the long length western blots were also presented in Supplementary Figure 4D), suggesting that an upregulation of PPARγ resulted in the priming of the protective anti-inflammatory switch.

### Optimal cell numbers for intra-arterial administration

Some experiments were performed to determine the optimal cell numbers for intra-arterial administration, considering that the purpose of this study was to investigate intra-arterial cell administration. As a result, the mortality of rats administrated with PBMCs via a common carotid artery depended on the cell numbers, as a large number of cells may result in embolic stroke (Supplementary Figure 5). The 20% mortality rate (0.21) associated with administration of intra-arterial PBMCs (5 × 10^5^ cells) was the lowest among the PBMC concentrations tested. Given these results, we administered 5 × 10^5^ cells per rats following the experiments to avoid death related to embolic stroke.

### Migration of administrated OGD-PBMCs into the brain parenchyma

**Figure 2:**
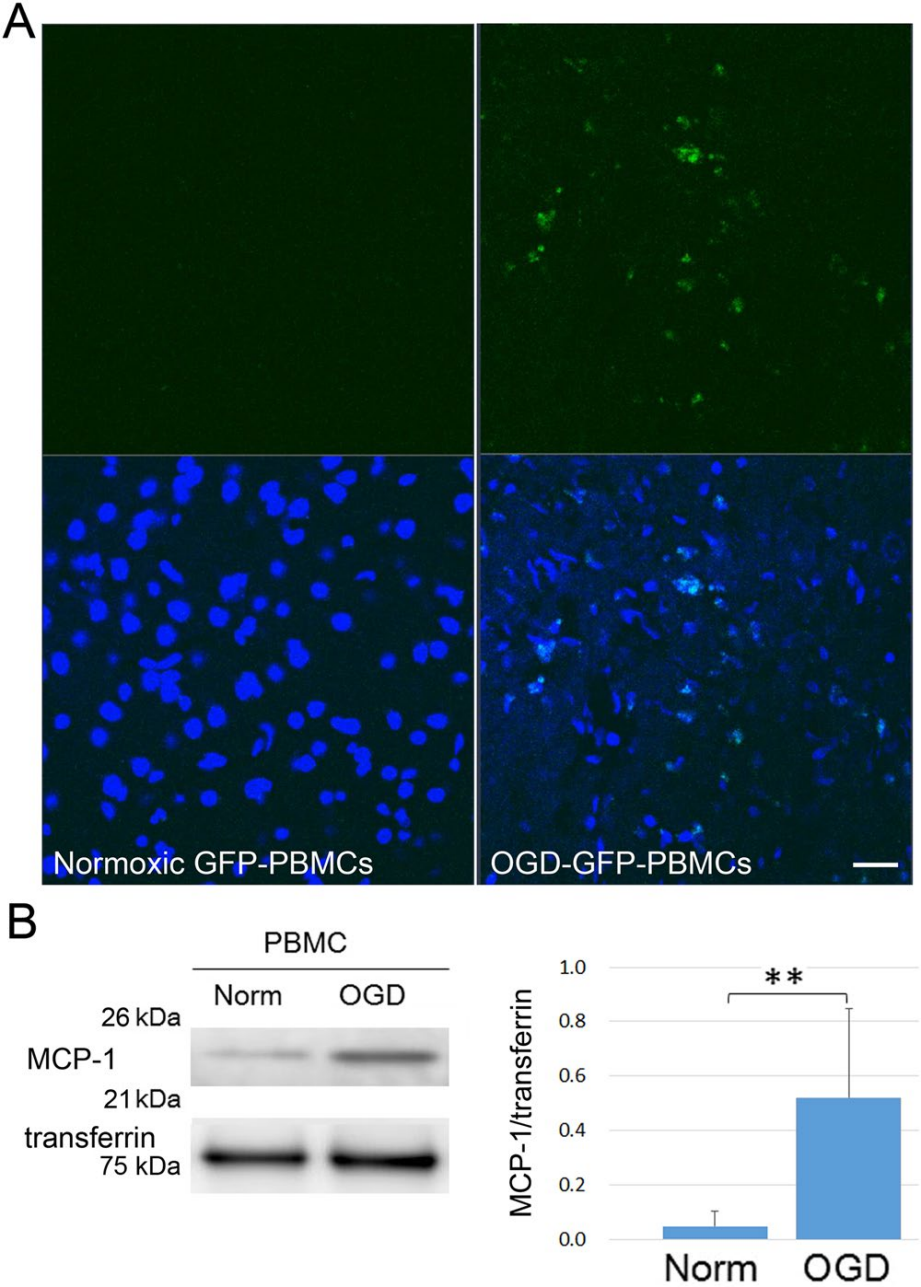
The migration of administrated OGD-PBMCs. (**A**) Administrated OGD-PBMCs from GFP mice (green, OGD-GFP-PBMCs) migrated into the border area between the ischemic core and the penumbra at 3 days after administration in triplicates. However, signals were not observed in the brain samples from administration of normoxic PBMCs from GFP mice (normoxic GFP-PBMCs). Scale bar, 50 μm. Analyses with 4′, 6′-diamidino-2-phenylindole (DAPI; blue) were performed by an investigator blinded to the therapeutic information. (**B**) Representative western blotting and bar graphs showing the relative signal intensities of monocyte chemoattractant protein-1 (MCP-1) from the conditioned media of rat primary-cultured PBMCs subjected to either normoxia (norm) or OGD (N = 5 each). The increase in the level of MCP-1 reveals that OGD-PBMCs might migrate in response to MCP-1 into the brain parenchyma. Transferrin confirmed an equal loading of proteins. Data represent relative optical densities of each sample compared to the loading control band by unpaired t-test. ^**^P < 0.01 (N = 5).

To confirm whether OGD-PBMCs migrated into the brain parenchyma across the BBB, both OGD-PBMCs and normoxic PBMCs derived from green fluorescent protein (GFP) transgenic mice^31^ were administered by an investigator blinded to the therapeutic information. Although OGD-GFP-PBMCs were observed in the border area between the ischemic core and the periphery at 3 days after administration (Fig. 2A), they were not found at 21 days after administration. However, signals were not observed in the normoxic GFP-PBMCs administration group.

Successively, the level of monocyte chemoattractant protein-1 (MCP-1), which is one of the key chemokines that regulates migration and infiltration into the brain lesions of monocytes/macrophages, PBMCs, and stem cells were investigated^26,32,33^. Additionally, MCP-1 deficiency was reported to decrease the susceptibility to infiltration and migration after cerebral ischemia^26,33^. To determine the effect of OGD precondition on MCP-1 secretion, the levels of MCP-1 on PBMCs under both the normoxic and the OGD conditions in the conditioned media were compared. The levels of MCP-1 under the OGD conditions were 6 times higher than those under the normoxic conditions (P = 0.006) (Fig. 2B).

### Increasing the number of SSEA-3-positive cells by OGD-PBMCs administration

**Figure 3:**
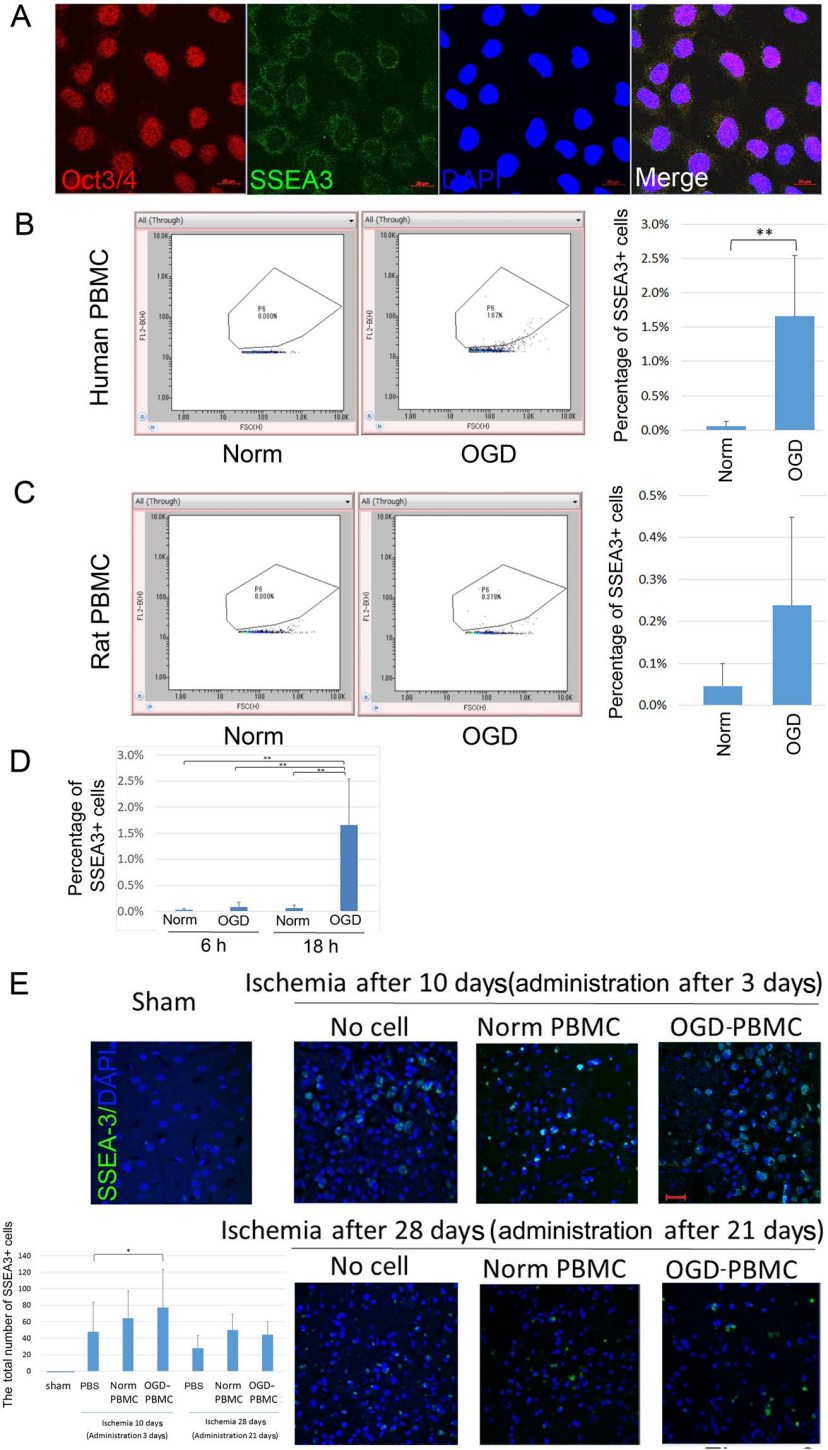
Increasing number of SSEA-3-positive cells after OGD *in vitro* and the presence of SSEA-3-positive cells after OGD-PBMCs administration *in vivo*. (**A**) Representative figures of octamer-binding transcription factor 3/4 (Oct3/4) (red)/stage-specific embryonic antigen-3 (SSEA-3) (green)/DAPI (blue) triple labelling of NTERA-2 cells, as examined by confocal microscopy. Scale bars, 20 μm. (**B, C**) Expression of SSEA-3 using both the human (**B**) and rat (**C**) OGD-PBMCs by flow cytometric analyses. The x-axis represents forward scatter and y-axis represents the SSEA-3-FITC channel. SSEA-3-positive cells in the OGD-PBMCs were more prominent than those in the normoxic PBMCs by unpaired t-test. **P < 0.01. (each N = 6). (**D**) The two point time series of expression of SSEA-3-positive cells in the human OGD-PBMCs by flow cytometric analyses by two-way ANOVA. (each N = 6). (**E**) SSEA-3-positive cell recruitment in the sham-operated and ischemic cerebral cortices at days 10 (i.e., administration after 3 days) and 28 (i.e., administration after 21 days) after ischemia. A bar graph represents the total number of SSEA-3-positive cells. Statistical analyses were performed by one-way ANOVA. A secondary-only antibody control confirms its specificity. Scale bars, 20 μm. *P < 0.05. (N = 28).

We speculated that OGD-PBMCs may increase the number of stem cells as SSEA-3-positive cells were detected in the PBMCs after cerebral ischemia^22^. Given that the positivity of anti-SSEA-3 antibodies was unknown, their positivity for stem cells was confirmed using pluripotent embryonal carcinoma cells (NTERA-2 cl.D1 [NT2/D1] (ATCC^®^ *CRL*-1973^™^), expressed SSEA-3^34^ and octamer-binding transcription factor 3/4 (Oct3/4) (Fig. 3A).

Subsequently, the expression of SSEA-3 in both human and rat OGD-PBMCs was evaluated by flow cytometric analyses using the anti-SSEA-3 antibody. The percentage of SSEA-3-positive cells in the OGD-PBMCs group was more prominent than that in the normoxic PBMCs group in human (P < 0.001) (Fig. 3B) as well as in rats (P = 0.07) (Fig. 3C). Actually, the number of SSEA-3-positive cells in the OGD-PBMCs group (2496 ± 1796/well) was more prominent than that in the normoxic PBMCs group (738 ± 996/well) in human (P = 0.037). Interestingly, increasing frequencies of SSEA-3-positive cells in OGD-PBMCs under the 18 h OGD condition were more apparent than those under 6 h OGD condition in human (P < 0.001) (Fig. 3D).

Finally, whether administration of OGD-PBMCs caused the increase in the number of SSEA-3-positive cells in the brain parenchyma after cerebral ischemia was investigated. Specifically, SSEA-3-positive cells were counted by immunofluorescence staining of the ischemic cortex at 3 and 21 days after administration (i.e., 10 and 28 days after cerebral ischemia, respectively) using the antibody against SSEA-3. Although SSEA-3-positive cells were not identified in the sham-operated group, they were observed in the ischemic core (Fig. 3E, Supplementary Figure 6). Lower magnification images qualitatively showed the overall status of the tissue (Supplementary Figure 6). However, it was difficult to discern quantitative differences among groups under low magnification. Seven randomly chosen non-overlapping high-power fields (630×) at the level of either the anterior commissure of the sham-operated or the ischemic cortex were examined (Supplementary Figure 7)^9,35,36^. The SSEA-3-positive cell numbers in the OGD-PBMCs administration group were more prominent than those in the phosphate buffered saline (PBS) control group at 3 days after administration (P = 0.04) (Fig. 3E). However, a difference in SSEA-3-positive cell numbers was not seen between the three groups at 21 days after administration. Additional experiments using different vendor’s antibody against SSEA-3 provided similar results as the ones mentioned above (Supplementary Figure 8).

### The expression of VEGF and TGF-β by OGD-PBMCs administration

**Figure 4:**
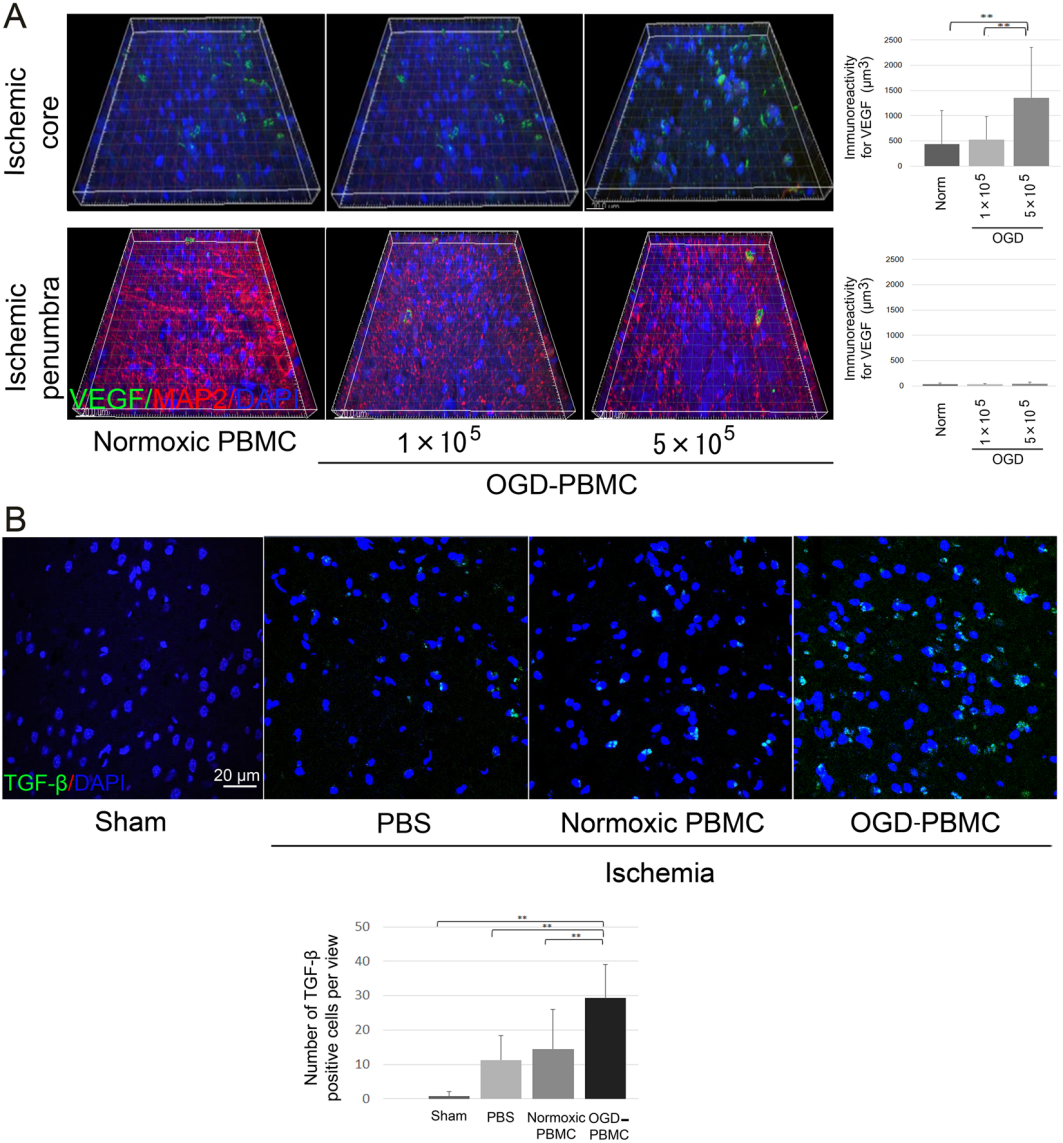
OGD-PBMCs administration promotes the expression of remodelling factors at 28 days after cerebral ischemia. Representative figures and the relative signal intensities of vascular endothelial growth factor (VEGF) (**A**) and transforming growth factor-β (TGF-β) (**B**) from the cerebral cortices of the transplanted groups at 28 days after cerebral ischemia. VEGF (**A**) and TGF-β (**B**) (green)/MAP2 (red)/DAPI (blue) triple labelling of the cerebral cortices in the border between the ischemic core and the penumbra at 28 days after cerebral ischemia, as examined by confocal microscopy. (**A**) A significantly higher immunoreactivity for VEGF was observed in the administration of the 5 × 10^5^ OGD-PBMCs group compared with the administration of the 1 × 10^5^ OGD-PBMCs and 5 × 10^5^ normoxic PBMCs administration in the border within the ischemic core (N = 28). In contrast, a difference in the immunoreactivity for VEGF was not observed in the ischemic penumbra. (**B**) A significantly higher number of TGF-β positive cells was observed in the administration of the OGD-PBMCs group compared with the administration of the normoxic PBMCs and PBS control groups in the border within the ischemic core (N = 21). Bar graphs represent the signal volume intensities of VEGF and the numbers of TGF-β positive cells of brain samples by one-way ANOVA. A secondary-only antibody control confirms its specificity. Scale bars, 20 μm. **P** < **0.01.

To confirm whether administration of OGD-PBMCs upregulates the remodelling factors in the injured brain parenchyma, immunohistochemical analyses of the brains of transplanted rats was performed using antibodies against both VEGF and TGF-β at 21 days after administration (i.e., 28 days after cerebral ischemia). While the expressions of VEGF and TGF**-**β were undetectable in the brains of sham-operated rats, marked expressions of VEGF and increasing numbers of TGF-β-positive cells were observed in the border area within the ischemic core at 21 days following administration (i.e., 28 days after cerebral ischemia) (Fig. 4A,B). Analyses of immunoreactivity intensities demonstrated that the administration of 5 × 10^5^ OGD-PBMCs caused more prominent expressions of VEGF compared to the administration of 5 × 10^5^ normoxic PBMCs and 1 × 10^5^ OGD-PBMCs (both P < 0.001) (Fig. 4A). In addition, the numbers of TGF-β-positive cells were more noticeable in the administration of OGD-PBMCs than those in the administration of normoxic PBMCs, while the same was not valid for the PBS control group (both P < 0.001) (Fig. 4B). However, differences between groups in the expression of VEGF and in the numbers of TGF-β-positive cells in the ischemic penumbra were not observed.

### Promotion of angiogenesis by administration of OGD-PBMCs

**Figure 5:**
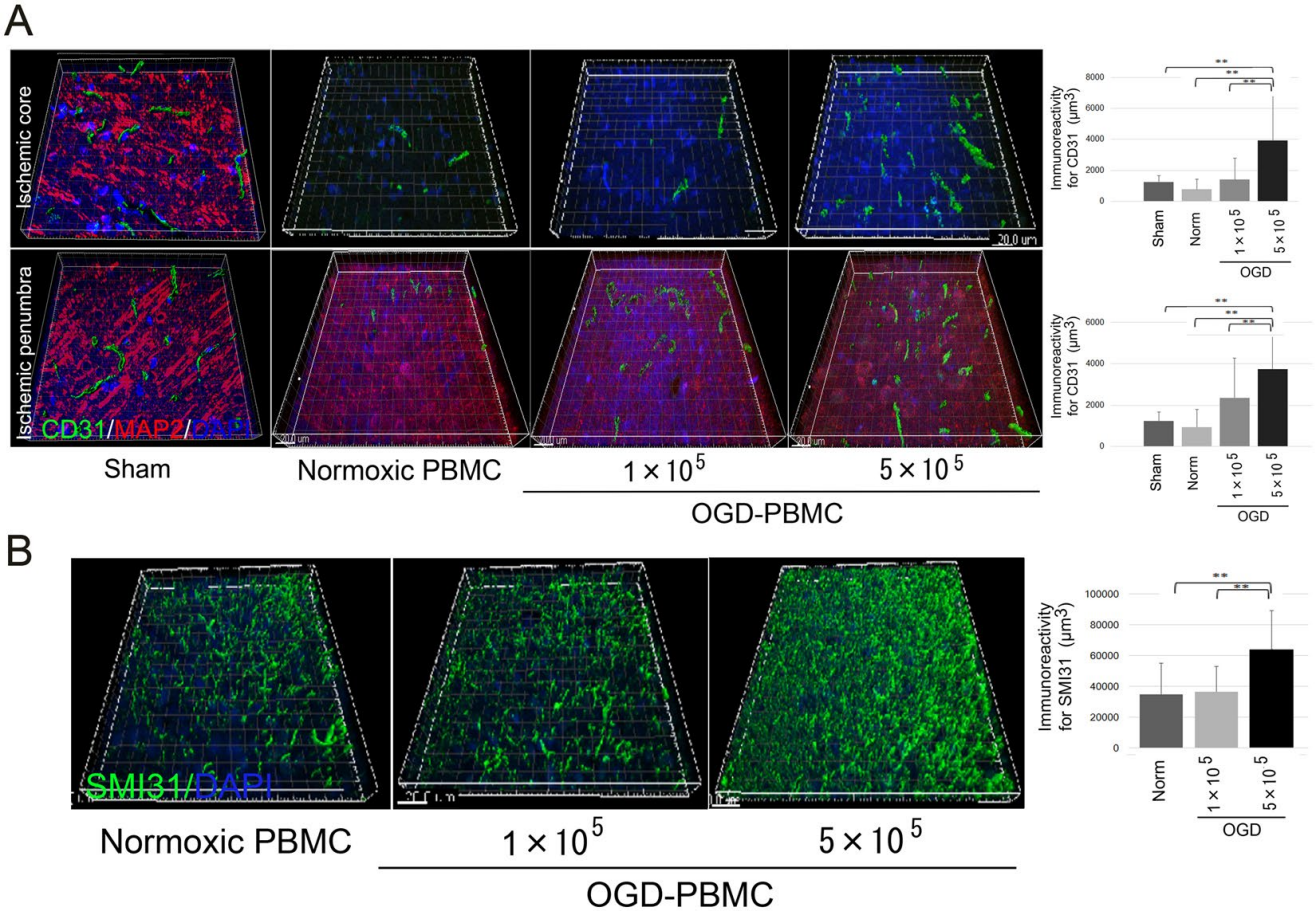
OGD-PBMCs administration promotes both angiogenesis in the border area between the ischemic core and the penumbra and axonal outgrowth in the ischemic penumbra at 28 days after cerebral ischemia. (**A**) Representative figures and bar graphs representing the immunoreactivity for cluster of differentiation 31 (CD31) volume, expressed as μm^3^, in the ischemic core and penumbra from the cerebral cortices of the sham-operated group, and administration of the normoxic PBMCs group, 1 × 10^5^ OGD-PBMC, or 5 × 10^5^ OGD-PBMC groups at 28 days after cerebral ischemia. The CD31 (green)/MAP2 (red)/DAPI (blue) triple labelling in the ischemic cortices at 28 days after cerebral ischemia, as examined by confocal microscopy. (**B**) Representative figures and bar graphs representing immunoreactivity for SMI31-positive volumes, expressed as μm^3^, in the ischemic penumbra from the cerebral cortices of the administration of the normoxic PBMCs, 1 × 10^5^ OGD-PBMCs, or 5 × 10^5^ OGD-PBMCs groups. SMI31 (green)/DAPI (blue) double labelling in the ischemic cortices at 28 days after cerebral ischemia, as examined by confocal microscopy (N = 28). The bar graph represents the signal volume intensities of CD31 and SMI31 of brain samples by one-way ANOVA. Moreover, a secondary-only antibody control confirmed its specificity. Scale bars, 20 μm. **P** < **0.01.

We speculated that the expression of both VEGF and TGF-β by administration of OGD-PBMCs might promote angiogenesis, given that our research group previously demonstrated that their expressions in the border area between the ischemic core and the penumbra by administration of OGD-microglia results in angiogenesis^9^. Therefore, the angiogenic effects of administration of OGD-PBMCs was assessed by immunofluorescence staining of the ischemic cortex at 28 days after cerebral ischemia using an antibody against the angiogenesis marker, i.e., CD31 (Fig. 5A). Confocal microscopic studies revealed that immunoreactivity of CD31 per unit volume in the border area between the ischemic core and the penumbra in the administration of 5 × 10^5^ OGD-PBMCs was more prominent than that found in the sham-operated group, and administration of normoxic PBMCs and 1 × 10^5^ OGD-PBMCs at 28 days after cerebral ischemia (i.e., at 21 days after administration) (all P < 0.001).

### Promotion of axonal outgrowth by administration of OGD-PBMCs

Furthermore, whether administration of OGD-PBMCs might prompt axonal outgrowth was investigated by immunofluorescence staining of the ischemic cortex at 28 days after cerebral ischemia using an antibody against the neurofilament protein marker, i.e., SMI31^9^. The immunoreactivity of SMI31 in the ischemic penumbra in the administration of 5 × 10^5^ OGD-PBMCs was more prominent than that in the administration of normoxic PBMCs and 1 × 10^5^ OGD-PBMCs at 28 days after cerebral ischemia (i.e., at 21 days after administration) (P < 0.001 and P < 0.001) (Fig. 5B).

### Therapeutic effects of the OGD-PBMCs administration against focal cerebral ischemia

**Figure 6:**
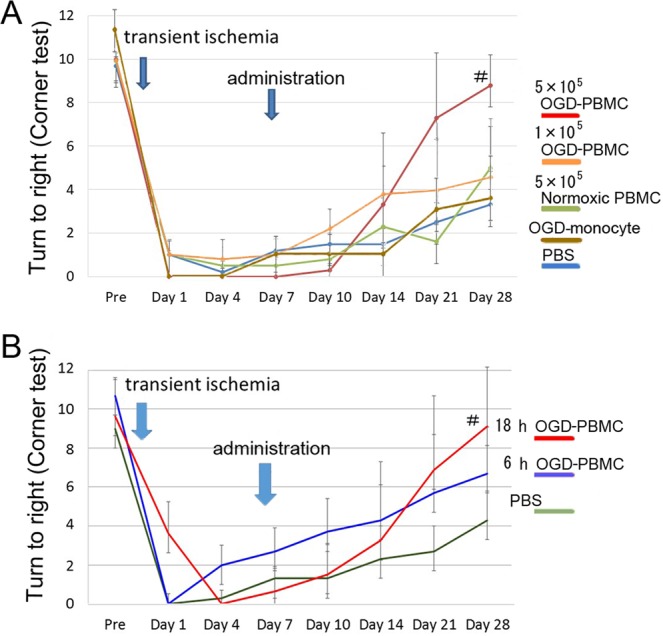
Improvements in the neurological outcomes after OGD-PBMCs administration. **(A)** A significantly better functional recovery as assessed in the corner test (performed 20 times) was observed in the administration of the OGD-PBMC group compared with the vehicle-injected control group (PBS control group) and normoxic PBMC group at 28 days after cerebral ischemia; ^#^P < 0.05 vs PBS control group (N = 6~8 per group). An absence of difference in functional recovery between the 5 × 10^5^ normoxic PBMCs administration group and the PBS control group was seen. In addition, no functional recovery was observed in the administration of both the 1 × 10^5^ OGD-monocytes (OGD-monocyte group) and 1 × 10^5^ OGD-PBMCs, and PBS control (N = 5~6) groups by two-way ANOVA. (**B**) A significantly better functional recovery as assessed in the corner test was observed in the administration of the 18 h OGD-PBMC group compared with the PBS control group. Finally, an improvement in the administration of the 6 h OGD-PBMC group was not found; ^#^P < 0.05 vs PBS control group by one-way ANOVA (N = 5~6).

Finally, whether OGD-PBMCs administration improved the functional outcome were investigated using a rat transient focal cerebral ischemia model. The neurological outcomes were compared between the PBS control group, normoxic PBMC administration group, and OGD-PBMC administration group. The neurological deficit, as measured by a sensorimotor scale with the corner test^37^, was significantly improved in the 5 × 10^5^ OGD-PBMC administration group compared to both the PBS control and 5 × 10^5^ normoxic PBMC administration groups at 28 days after cerebral ischemia (P = 0.020 and 0.268, respectively) (Fig. 6A). In addition, the delivery routes for cell administration were evaluated. An improvement in the rats administrated with 5 × 10^5^ OGD-PBMCs via a femoral vein was not found (Supplementary Figure 9). Additionally, allergic reactions, symptoms of graft versus host diseases, intracerebral haemorrhage, and oedema were not seen after administration.

Successively, to investigate the functional recovery by cell number-dependence of administration of OGD-PBMCs, the neurological outcomes between the groups with administrations of 5 × 10^5^ and 1 × 10^5^ OGD-PBMCs were analysed. The neurological deficit, as measured by the corner test, was significantly improved in the group with an administration of 5 × 10^5^ OGD-PBMCs compared with the PBS control groups (P = 0.028) at 28 days after cerebral ischemia (i.e., at 21 days after administration), although the group with an administration of 1 × 10^5^ OGD-PBMCs did not show significant improvements compared with the PBS control group (P = 0.497) (Fig. 6A).

### Adequate conditions of the OGD-PBMCs administration

To determine whether administration of OGD-PBMCs or isolated monocytes/macrophages preconditioned by OGD improved the functional outcome, the therapeutic effects of isolated monocytes/macrophages preconditioned by OGD were evaluated. Given that the frequency of monocytes/macrophages is generally about 20~40% in PBMCs^18^, 1 × 10^5^ monocytes/macrophages preconditioned by OGD were administered. Following intra-arterial administration of these cells, an improvement at 28 days after cerebral ischemia (i.e., at 21 days after administration) was not observed (Fig. 6A).

Finally, to investigate the adequate duration of OGD, the neurological outcomes between the 6 and 18 h OGD-PBMCs administration groups were compared through the corner test after focal cerebral ischemia. Although the 18 h OGD-PBMCs administration group improved compared with the PBS control group (P = 0.028), the 6 h OGD-PBMCs administration group reported similar results to the PBS control group (P = 0.69) (Fig. 6B).

## Discussion

**Figure 7:**
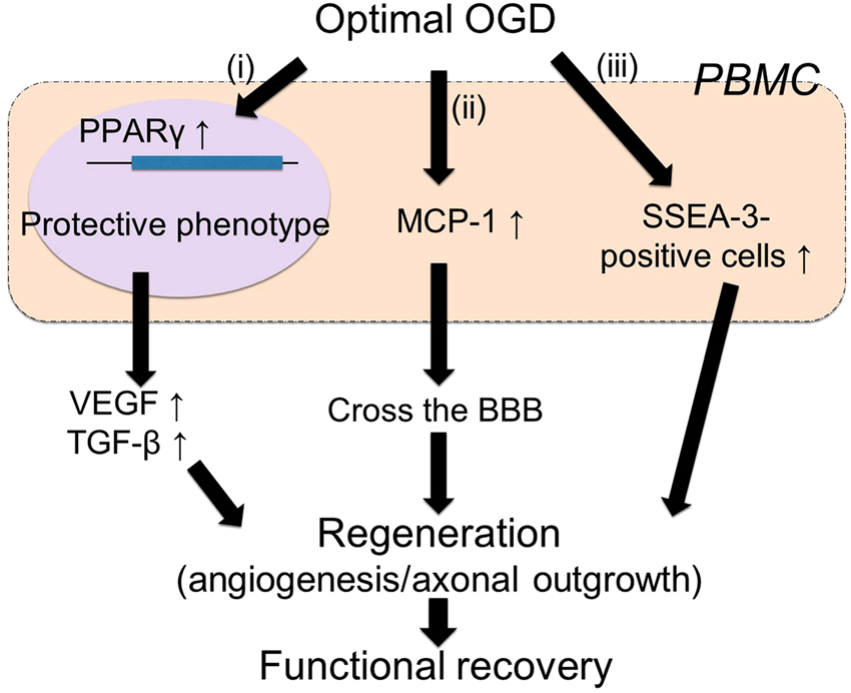
Mechanism of OGD-PBMC administration after cerebral ischemia. The schema of protective polarization of PBMCs and a diagram of therapeutic effects of OGD-PBMC administration after cerebral ischemia. (i) The optimal OGD condition upregulate transcription factor PPARγ in PBMCs, which mediates such a monocyte/macrophage polarisation from the pro-inflammatory to the protective phenotype. (ii) The OGD condition induced PBMCs to increase the secretion of both VEGF and TGF-β *in vitro*. Moreover, the OGD condition upregulated MCP-1 to be able to cross the BBB. In addition, (iii) the OGD condition stimulated to increase numbers of SSEA-3-positive PBMCs. These effects of OGD-PBMC might induce regeneration such as angiogenesis and axonal outgrowth. Finally, administration of OGD-PBMCs might result in functional recovery.

The following therapeutic mechanisms by administration of OGD-PBMCs were considered to be involved in: (i) the protective switch in PBMCs preconditioned by the optimal OGD condition, (ii) the migration of OGD-PBMCs into the periinfarct area, and (iii) the increase in the number of SSEA-3-positive cells (Fig. 7).

Firstly, that the ability of the optimal OGD condition to lead to the protective switch in the PBMCs phenotype was demonstrated. In fact, given that the 18 h OGD was previously shown to cause the protective switch in primary microglia^9^, the OGD condition induced PBMCs to increase the secretion of both VEGF and TGF-β *in vitro*, indicating a polarisation into the protective phenotype (Fig. 1). Considering that the expression of both VEGF and TGF-β in the isolated monocytes under this OGD condition was not detected, synergistic effects between monocytes and other cells, such as the T cells and/or other mononuclear cells in the PBMCs, might be required for the upregulation of VEGF and TGF-β. In addition, the OGD condition was confirmed to upregulate PPARγ, which mediates such a monocyte/macrophage polarisation from the pro-inflammatory to the protective phenotype^30^. We speculate the pleiotropic effects of OGD-PBMCs, which include the paracrine actions of the remodelling factors, to promote angiogenesis and axonal outgrowth^9,37–40^ (Fig. 7(i)). Administrated OGD-PBMCs directly secreted VEGF and TGF-β, although administrated OGD-PBMCs were not observed at 21 days after administration. These factors may also be associated with changes in resident native endothelial cells, astrocytes, pericytes, neurons, and microglia caused by the paracrine action of OGD-PBMCs like OGD-microglia^9^. Several studies have indicated both angiogenesis and axonal outgrowth to be a necessary functional recovery after cerebral ischemia^38–40^. These results suggested that the optimal OGD condition could lead to the protective switch in the PBMCs phenotype through the upregulation of the PPARγ transcription factor and prompt tissue regeneration.

Secondly, OGD-PBMCs, but not normoxic PBMCs, were found to be able to cross the BBB to reach the injured brain parenchyma, although several reports described the primary monocytes/macrophages and PBMCs without stimuli to cross the BBB and reach the injured brain parenchyma^15,20,26^. We speculated that MCP-1 upregulation resulted in such an ability to cross the BBB by OGD-PBMCs compared to normoxic PBMCs (Fig. 7(ii)). Thus, this OGD-PBMCs ability is more crucial than that of normoxic PBMCs to the success of cell therapies.

Thirdly, the increasing numbers of cells with the pluripotent surface marker, i.e., SSEA-3, may be one of therapeutic mechanisms for the administration of OGD-PBMCs. In fact, stem cells positive for SSEA-3 were initially identified by applying stress to mesenchymal stem cells and were termed multilineage differentiating stress-enduring cells (Muse cells)^27^. Additionally, administration at the delayed subacute phase of cerebral ischemia showed that Muse cells facilitated the neural reconstruction and improved functions^6,28^. We demonstrated such increasing numbers of SSEA-3-positive PBMCs under the optimal OGD conditions *in vitro* and after their administration *in vivo* (Fig. 3). Whether a small number of SSEA-3-positive PBMCs play a significant role after cerebral ischemia is yet to be fully defined. The mechanisms underlying an increase in OGD-PBMCs and in the cerebral ischemic lesion remain unclear; however, it improves the functional outcome (Fig. 7(iii)).

OGD-PBMCs are a practical and convenient cell source for cell therapies. Cell therapies using embryonic stem cells^41^, or induced pluripotent stem cells^42^ were also prompted functional recovery after ischemic stroke in animal models. However, the use of embryonic stem cell involves an ethical problem, and the tumorigenic potential of induced pluripotent stem cells is a major safety concern for clinical translation^42^. An experimental model of cerebral hypoxia-ischemia reported that PBMCs administration without any stimulations improved the outcome, even though the mechanism behind the functional recovery was yet not known^20^. Our idea using OGD-PBMCs is superior to the previous one considering the protective switch. In addition, a patient treated with multiple injections of allogeneic stem cells from different sources against ischemic stroke developed a glioproliferative lesion, which resulted in paraplegia and which ultimately required radiotherapy^43^. With regards to the immunological concerns, autologous cells are safer than allogenic cells. Moreover, isolation and preparation of autologous PBMCs are established techniques. Our results are therefore very promising for a clinical application.

This OGD-PBMCs technique might be a potential candidate for therapeutic applications in ischemic stroke, given its attractive protective functions and simplicity for clinical application. Therefore, further clinical research towards the development of innovative OGD-PBMCs therapies should be conducted. In conclusion, OGD-PBMCs administration was identified to be a novel therapeutic strategy for ischemic stroke.

## Methods

This study was conducted in strict accordance with the recommendations from the Guide for the Care and Use of Laboratory Animals of the National Institutes of Health (Bethesda, MD, USA). The protocol (#SD00931) was approved by the Niigata University Administrative Panel on Laboratory Animal Care and the Ethical Committee of Niigata University. The ethical approval for the present study (#2017–0020) was also provided by the Institutional Ethics Committee of the Niigata University Medical and Dental Hospital. All the surgeries were performed under inhalation of isoflurane and according to the ARRIVE (Animal Research: Reporting of *In Vivo* Experiments) guidelines^44^. Rats and mice were maintained under controlled light (lights on, 5:00–19:00), temperature (23 ± 1 °C), and humidity (55 ± 10%) conditions and given free access to food and water^9,36,45^.

### Primary cell cultures

PBMCs were obtained using the Ficoll-Paque centrifugation (GE Healthcare, 17–5446–02), according to the manufacturer’s instructions. Primary monocytes were isolated from the PBMCs by MACS CD11b (Miltenyi Biotec, 130-049-601).

To investigate the secretion of VEGF from PBMCs after OGD, the conditioned media from PBMCs was used. Briefly, after OGD was performed using primary PBMCs, the level of VEGF in the conditioned media was measured using the human VEGF Quantikine® ELISA Kit (DVE00, R&D Systems, Minneapolis, MN, USA) and the mouse VEGF Quantikine® ELISA Kit (RRV00, R&D Systems)^45^, according to the manufacturer’s instructions (N = 4~6).

### Oxygen–glucose deprivation

The standardised conditions for OGD were described in detail elsewhere^9,45^. The cultures containing a low-glucose medium were placed in a hypoxia chamber (Billups-Rothenburg, Del Mar, CA, USA), which was first flushed with a mixture of 95% N_2_ and 5% CO_2_ for 1 h and then closed for 6, 18 or 30 h^9,45^.

### Western blotting

For the whole-cell extracts *in vitro*, cells were harvested in cold RIPA buffer (ThermoFisher Scientific, Waltham, MA, USA) and the conditioned media were collected. Proteins in the samples were separated by tris-glycine SDS-PAGE and were probed with primary antibodies (Supplementary Table 1), followed by a secondary horseradish peroxidase-conjugated antibody. Signals were detected by an enhanced chemiluminescence (GE Healthcare) and semi-quantified by densitometry. Finally, membranes were stripped and probed with either an anti-β-actin or an anti-transferrin antibody to confirm the even loading of proteins.

### Focal cerebral ischemia

Transient focal cerebral ischemia was induced in male Sprague-Dawley rats weighing 290–320 g using an intraluminal filament suture technique^36,46^. Thereafter, after 90 min of ischemia, the suture was withdrawn to restore the blood flow. This model shows an area of the ischemic core and penumbra determined by the presence of microtubule associated protein 2 (MAP2) with a high degree of reproducibility^36,46^. Additionally, it demonstrates that the time window for the salvage of the penumbral tissues by reperfusion was 90 min. Finally, the core body temperature, which was monitored via a rectal probe, was maintained at 37.0 ± 0.5 °C using a heating pad.

### Cell administration

Cells were diluted with 200 μL of PBS^9,11^. Rats subjected to transient MCA occlusion at 7 days after cerebral ischemia were randomly assigned to one of the cell-treated groups, in which administration of cells was slowly infused through the stump of the external carotid artery over 3 min (cells group), the same was not valid for the PBS control groups^9,11^. The same volume of PBS was injected in all groups.

### Experimental design

Sample size calculations were performed prior to the experiments to determine the number of animals required to detect differences between the cell administration and control conditions. Based on a pilot study of N = 4 animals in the treatment group, we determined the sample size needed to detect differences in the motor outcomes between the administration of OGD-PBMCs and normoxic PBMCs, and the PBS control group (α, 0.05; one-sided analysis). Ischaemic rats (N = 57) were randomly assigned to various intra-arterial administration groups —5 × 10^5^ normoxic PBMCs group (N = 6; mortality rate, 0.33), 1 × 10^5^, OGD-PBMCs group (N = 13; mortality rate, 0.23), 5 × 10^5^ OGD-PBMCs group, (N = 14; mortality rate, 0.21), PBS group (N = 13; mortality rate 0), 6-h 5 × 10^5^ OGD-PBMCs group (N = 5; mortality rate 0), and 5 × 10^5^ OGD-PBMCs intra-venous administration group (N = 6, mortality rate 0), while the analyses were performed by an investigator blinded to the therapeutic information.

### Immunofluorescence staining and confocal microscopy

Rats that survived for 10 and 28 days after cerebral ischemia were euthanised with an overdose of isoflurane, followed by transcardial perfusion with normal saline and by perfusion with cold 4% paraformaldehyde in a 0.1 M PBS (pH 7.4). Thereafter, brains were removed and embedded in paraffin wax, while serial sections (4-μm thick) were then cut from the paraffin blocks and stained using antibodies, as previously described^36,45,47^. Furthermore, free-floating sections (50-μm thick)^36^ were stained and mounted with Vectashield 4′, 6′-diamidino-2-phenylindole (DAPI) (Vector Laboratories, Burlingame, CA, USA). Further information about the primary antibodies are provided in Supplementary Table 1. Subsequently, the sections were examined under a confocal two-photon-scanning microscope (LSM710; Carl Zeiss, Oberkochen, Germany), whereas cortical tissues corresponding to either the ischemic core or penumbra were defined by MAP2 staining, as described elsewhere^36,46^.

### Green fluorescent protein (GFP) mice

To determine whether transplanted PBMCs can translocate from the blood into the brain parenchyma to exert their beneficial effects after intra-arterial administration, PBMCs from GFP mice were used^31^. GFP transgenic mice were produced by breeding heterozygous pairs in the Genome Information Research Centre, Osaka University, Japan and maintained in the Department of Comparative and Experimental Medicine, Brain Research Institute, Niigata University. Following the preconditioning of primary PBMCs from GFP mice by OGD, these cells were administered intra-arterially at 7 days after cerebral ischemia. Finally, a confocal microscopic examination was conducted at 3 and 21 days after administration in triplicates^9^.

### Quantitative analysis of brain tissue structures by immunostaining

To perform quantitative analyses of the brain tissue structures, tissue sections were immunostained with antibodies against CD31 (i.e., a marker of endothelial cells and angiogenesis), MAP2 (i.e., a marker of neuronal dendrites), SMI31 (i.e., a marker of neuronal axons), VEGF and TGF-β (Supplementary Table 1), and counted as previously described^9,47^. Briefly, seven randomly chosen non-overlapping high-power fields (630 × ) at the level of either the anterior commissure of the sham-operated or the ischemic cortex in the MCA territory were examined. Data were acquired from stereotaxically identical 0.03-mm^3^ regions of interest (ROIs). Successively, three-dimensional reconstructions and z-sections collected at 0.15-μm z-intervals were created and automatically quantified in a blinded fashion as the intensity of the total immunoreactive structure volumes (immunoreactive volume/examined ROI volume) using the IMARIS imaging software (BitplaneAG, Zurich, Switzerland)^9,47–49^. Finally, the results were confirmed in either three or four independent samples (N > 21–28).

### Cell counting protocol

To determine the frequency of cells positive for SSEA-3 and TGF-β, seven randomly chosen non-overlapping high-power fields (630×) at the level of either the anterior commissure of the sham-operated or the ischemic cortex (i.e., the border area between the ischemic core and the penumbra) were examined at 10 and 28 days after cerebral ischemia (N = 21)^35,36^.

### Flow cytometric analysis

PBMCs were gated and analysed by a flow cytometer (On-chip Biotechnologies Co., Ltd, Japan). While 2500 cells were analysed in each experiment of PBMCs, the data were assessed using the On-Chip Flow version 1.7.9 program.

### Sensorimotor assessment

Sensorimotor assessments were performed at 0, 1, 4, 7, 10 (i.e., 3 days after administration), 14 (i.e., 7 days after administration), 21 (i.e., 14 days after administration), and 28 days (i.e., 21 days after administration) after cerebral ischemia using the corner test^9,33^. Analyses of the therapeutic effects were conducted by an investigator blinded to the therapeutic information.

### Statistical analyses

All data are presented as the mean ± standard deviation (SD). Differences in the parameters were evaluated using either a one-way or a two-way ANOVA followed by either the Dunnett or Bonferroni’s *post hoc* tests or the unpaired *t*-test. All statistical analyses were performed using IBM SPSS Statistics for Windows, Version 25.0 (Armonk, NY, USA). All tests were considered statistically significant at a P value < 0.05.

## Supplementary information

Supplementary information
