# Publisher Correction: A novel therapeutic approach using peripheral blood mononuclear cells preconditioned by oxygen-glucose deprivation

**DOI:** 10.1038/s41598-019-55308-2

**Published:** 2019-12-20

**Authors:** Masahiro Hatakeyama, Masato Kanazawa, Itaru Ninomiya, Kaoru Omae, Yasuko Kimura, Tetsuya Takahashi, Osamu Onodera, Masanori Fukushima, Takayoshi Shimohata

**Affiliations:** 1 Department of Neurology, Brain Research Institute, Niigata University, 1-757 Asahimachi-dori, Chuoku, Niigata, 951-8585 Japan; 2 Translational Research Center for Medical Innovation, Foundation for Biomedical Research and Innovation at Kobe, 2-2 Minatojima-Minamimachi, Kobe, 650-0047 Japan; 3 Department of Neurology, Gifu University Graduate School of Medicine, 1-1 Yanagido, Gifu, 501-1194 Japan

Correction to: *Scientific Reports* 10.1038/s41598-019-53418-5, published online 14 November 2019

In the original version of this Article, Masahiro Hatakeyama was incorrectly listed as a corresponding author. The correct corresponding authors for this Article are Masato Kanazawa and Takayoshi Shimohata. Correspondence and request for materials should be addressed to masa2@bri.niigata-u.ac.jp or shimo@gifu-u.ac.jp. This error has now been corrected in the HTML and PDF versions of the Article.

