# Supplementary material for: A novel therapeutic approach using peripheral blood mononuclear cells preconditioned by oxygen-glucose deprivation
Source: Sci Rep. 2019 Nov 14;9:16819. doi: 10.1038/s41598-019-53418-5 (PMC6856386; doi:10.1038/s41598-019-53418-5)
Supplement: Supplementary file 1 — Supplementary information [file 41598_2019_53418_MOESM1_ESM.pdf]

A novel therapeutic approach using peripheral blood mononuclear cells preconditioned by oxygen-glucose deprivation

Masahiro Hatakeyama, M.D.,[1] Masato Kanazawa, M.D., Ph.D.,[1] Itaru Ninomiya, M.D.,[1] Kaoru Omae, M.A.,[2], Yasuko Kimura, Ph.D.,[2], Tetsuya Takahashi, M.D., Ph.D.,[1] Osamu Onodera, M.D., Ph.D.,[1] Masanori Fukushima, M.D., Ph.D.,[2] and Takayoshi Shimohata, M.D., Ph.D.[3]

[1]Department of Neurology, Brain Research Institute, Niigata University, 1-757 Asahimachi-dori, Chuoku, Niigata 951-8585, Japan

[2]Translational Research Center for Medical Innovation, Foundation for Biomedical Research and Innovation at Kobe, 2-2 Minatojima-Minamimachi, Kobe 650-0047, Japan

[3]Department of Neurology, Gifu University Graduate School of Medicine, 1-1 Yanagido, Gifu 501-1194, Japan

Supplementary Table 1

Antibodies	Source	Dilutions	Appl	Manufacturer	Catalog #
CD31	Rat	1:20	IF	Dianova	DIA-310
CD206	Gout	1:250	IF	R&D Systems, Inc	AF2523
iNOS	Rabbit	1:50	IB	Abcam	ab15323
Mac-1 (CD11b/CD18)	Rat	1:1000	flow cytometry	Abcam	ab24874
MAP2	Mouse	1:250	IF	Sigma-Aldrich	MM9942
MCP-1	Rabbit	1:2000	IB	Abcam	ab7202
Oct3/4	Mouse	1:100	IF	Santa Cruz Biotechnologies	sc-5279
PPARγ	Rabbit	1:500	IB	Abcam	ab59256
SMI31	Mouse	1:500	IF	ConvanceBio Legend	801601SMI-31R
SSEA-3	Rat	1:500/1:100	IF/flow cytometry	STEMCELL technologies	60061AD
SSEA-3	Rat	1:100	IF	BD biosciences	560236
TGF-β	Rabbit	1:500/1:2000	IB/IF	Torrey Prince Biolabs	TP254
TNF-α	Rat	1:500	IB	Peprotech	500-P72
VEGF	Rabbit	1:100	IB	Abcam	ab46154
VEGF	Rabbit	1:200	IF	Santa Cruz Biotechnologies	sc-152
β-actin	Gout	1:2000	IB	Santa Cruz Biotechnologies	sc-1616
transferrin	Rabbit	1:500	IB	Abcam	ab82411

Supplementary Table 1. Information of primary antibodies used in the present study.

Appl, Application; CD11b, cluster of differentiation 11b; CD18, cluster of differentiation 18; CD31, cluster of differentiation 31; CD206, cluster of differentiation 206; IB, immunoblotting; IF, immunofluorescence; iNOS, inducible nitric oxide synthase; Mac-1, macrophage-1; MAP2, microtubule-associated protein 2; MCP-1, monocyte chemotactic protein-1; Oct3/4, octamer-binding transcription factor 3/4; SSEA-3, stage-specific embryonic antigen 3; TGF-β, transforming growth factor beta; TNF-α, tumour necrosis factor alpha; VEGF, vascular endothelial growth factor.

Supplementary Figure 1

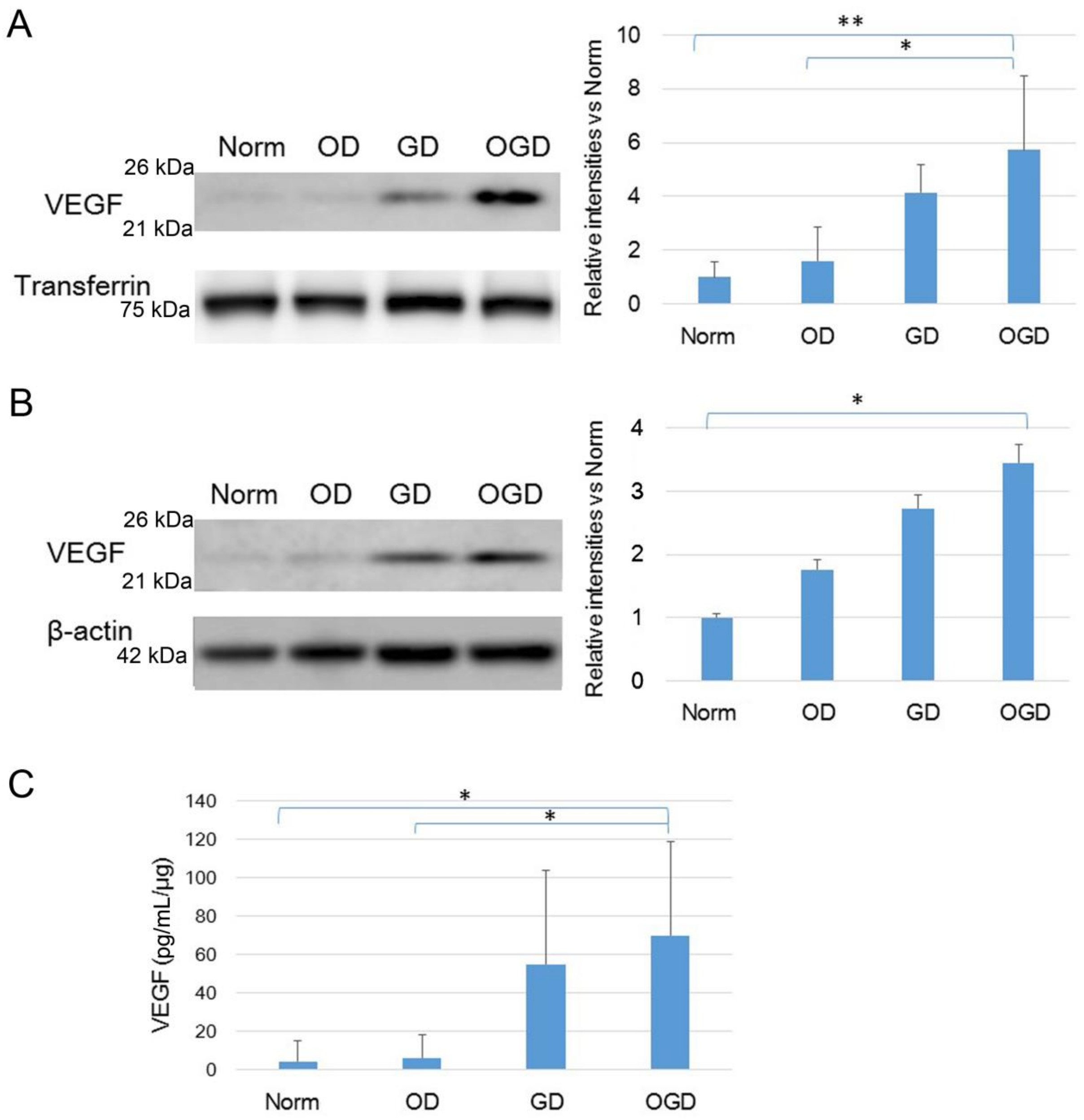

Supplementary Figure 1. Oxygen-glucose deprivation (OGD) condition to secrete VEGF from human PBMCs is superior to glucose deprivation alone (GD), oxygen deprivation alone (OD), and normoxic condition.

Representative western blotting and optical densitometric analyses from conditioned media (A) and cell lysates of human PBMCs (B) for VEGF 18 h after stimulation. Data represent relative optical densities of OD, GD, and OGD condition samples compared with those of normoxic condition samples (both N = 6). Transferrin and β-actin confirmed equal loading of proteins. (C) Enzyme-linked immunosorbent assay demonstrates that the level of VEGF after 18 h incubation under OGD from human PBMC conditioned media (70.0 ± 49.2 pg/mL/μg) was higher than that from PBMC conditioned media under OD (6.2 ± 12.0 pg/mL/μg) (P = 0.015) and normoxic condition (4.3 ± 10.6 pg/mL/μg) (P = 0.012) (N = 6). Statistical analyses were performed by one-way ANOVA. **$P < 0.01$, *$P < 0.05$.

Supplementary Figure 2

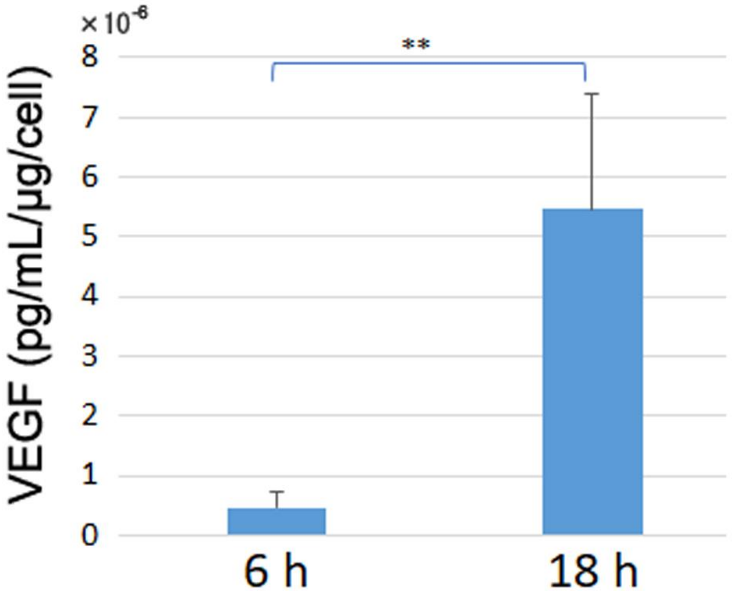

Supplementary Figure 2. The 18-h OGD condition was superior to the 6-h OGD condition for VEGF secretion in PBMCs.

Enzyme-linked immunosorbent assay demonstrates that the VEGF level in rat PBMC-conditioned media after 18-h incubation under OGD ($5.5 \pm 2.0 \times 10^{-6}$ pg/mL/μg/cell) was higher than that in PBMC-conditioned media under 6-h OGD ($0.5 \pm 0.3 \times 10^{-6}$ pg/mL/μg/cell) ($P < 0.001$) ($N = 4$). Statistical analyses were performed using the unpaired t-test. **$P < 0.001$.

Supplementary Figure 3

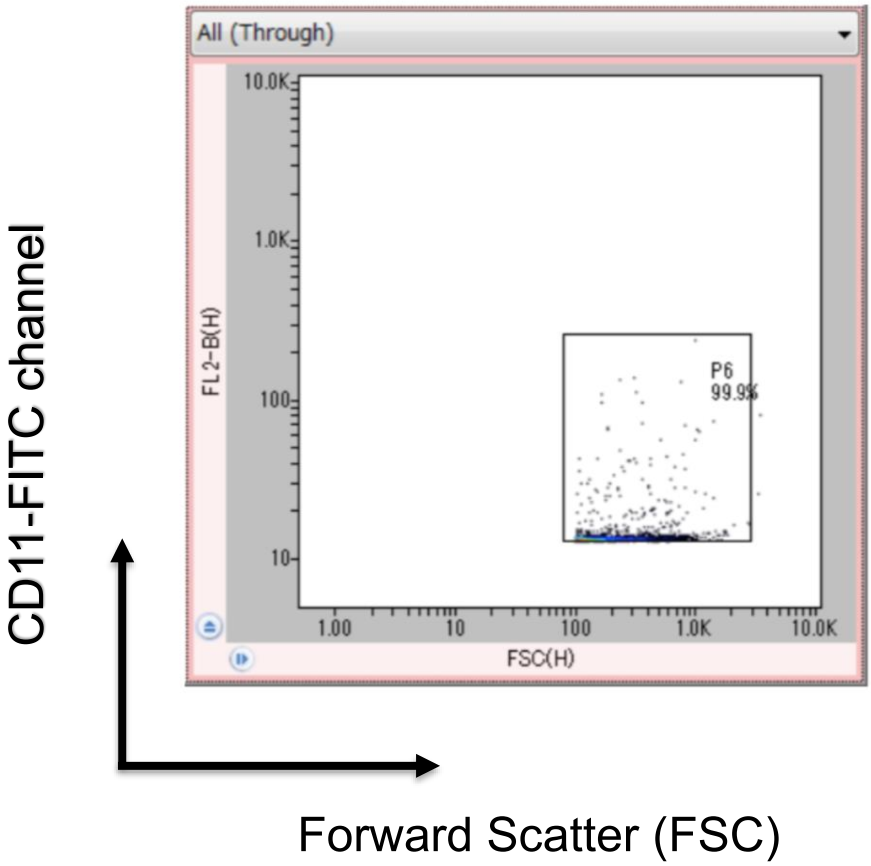

Supplementary Figure 3. Isolation of monocytes using a magnetic cell sorting (MACS) technique.

After isolation using MACS, the gated cells that were positive for CD11b, a marker of monocytes/macrophages, was more than 99% in triplicate experiments.

Supplementary Figure 4

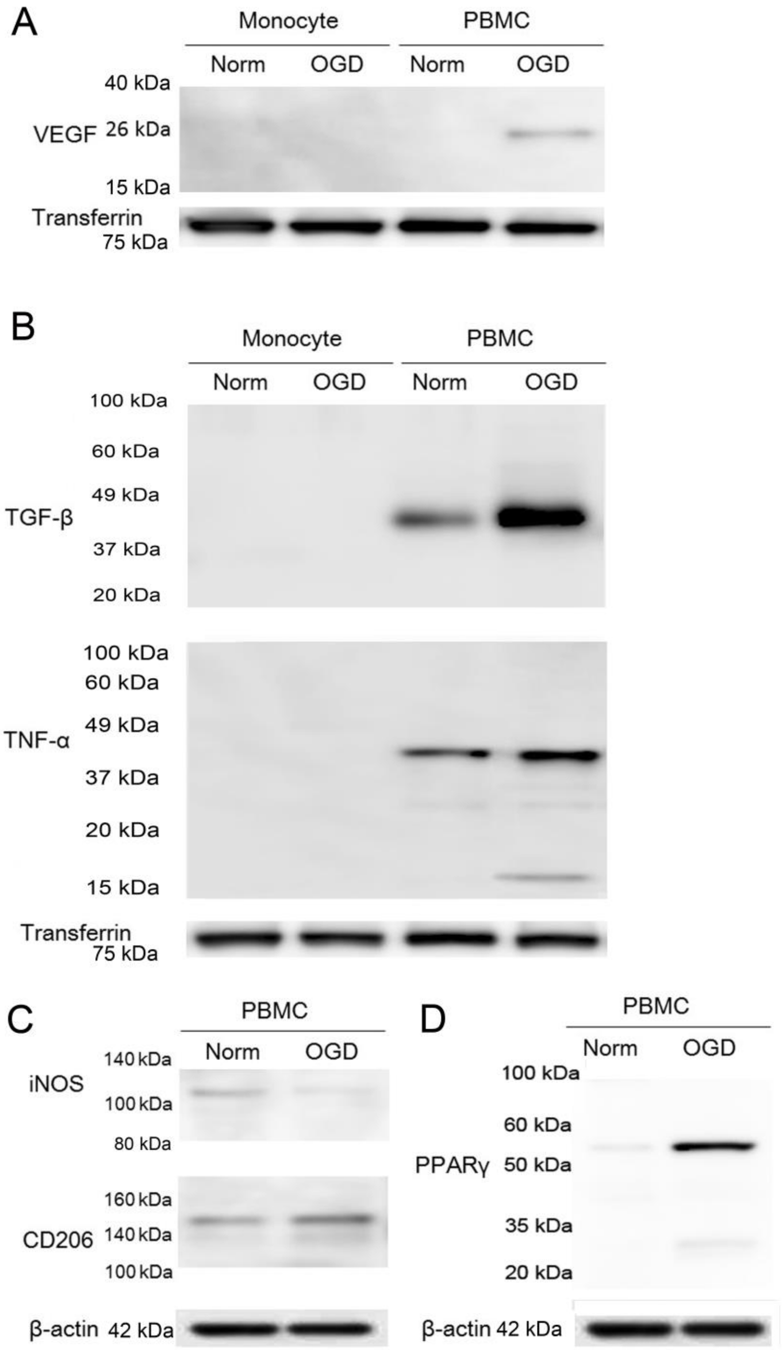

Supplementary Figure 4. Characteristics of rat primary-cultured OGD-PBMCs.

The long length western blots shown in the main manuscript Figure 1 were presented. The secretory vascular endothelial growth factor (VEGF) **(A),** anti-inflammatory cytokine transforming growth factor-β (TGF-β) and pro-inflammatory cytokine tumour necrosis factor-α (TNF-α) **(B)** from the conditioned media of rat primary-cultured PBMCs and isolated monocytes subjected to either normoxia (norm) or OGD. (**C**) Representative figures indicate the expression of the pro-inflammatory marker (i.e., inducible nitric oxide synthase, iNOS) and the protective anti-inflammatory marker (cluster of differentiation 206, CD206) in the cell lysate from PBMCs under both the normoxic and the OGD conditions. (**E**) Representative figures indicate the expression of the peroxisome proliferator-activated receptor γ (PPARγ) in the cell lysate from PBMCs under both the normoxic and the OGD conditions. Transferrin and β-actin confirmed an equal loading of proteins.

Supplementary Figure 5

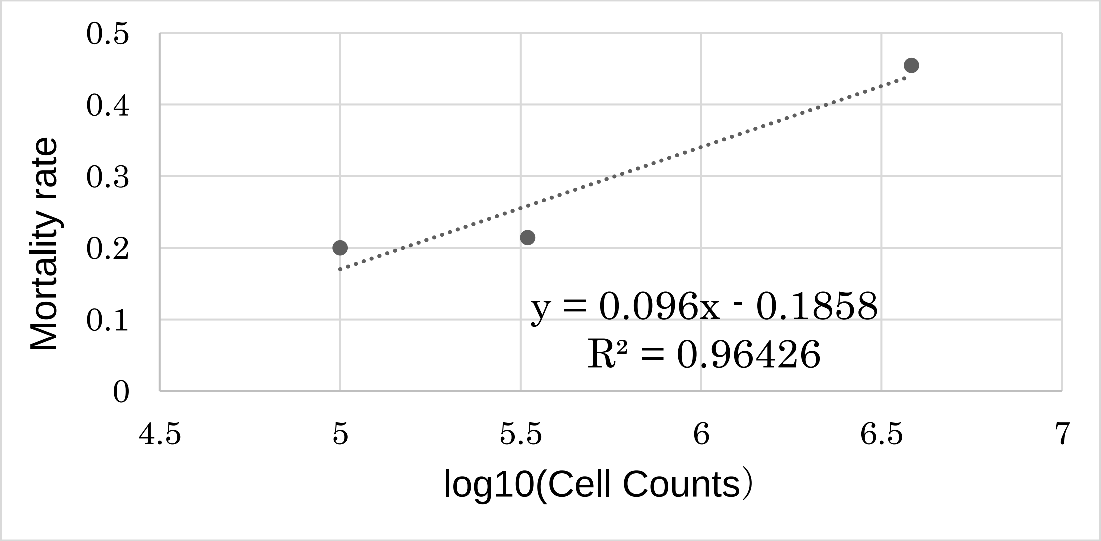

Supplementary Figure 5. Operation-related mortality rate after peripheral blood mononuclear cell (PBMC) administration via intra-arterial route.

Administration of too much cells ($10^{6.5}$ PBMCs) resulted in death after operations ($10^{6.5}$ PBMCs ; N = 11, $10^{5.5}$ PBMCs; N = 14, 10^5 PBMCs; N = 13). Administration of 10^5 and $10^{5.5}$ PBMCs were low mortality rate among these cell numbers.

Supplementary Figure 6

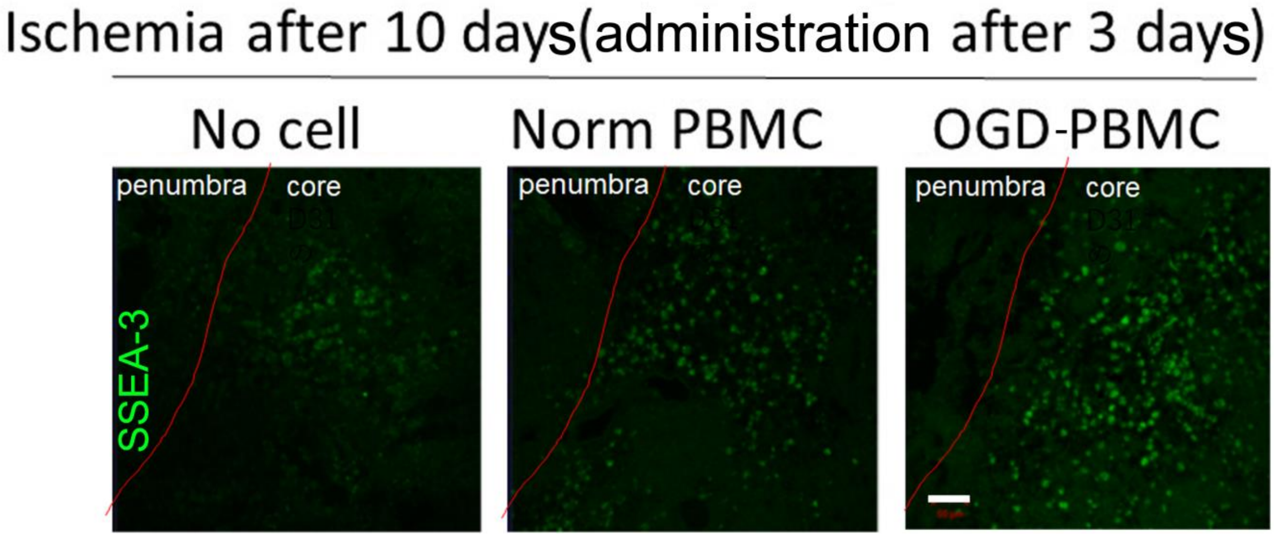

Supplementary Figure 6. Increasing number of SSEA-3-positive cells 3 days after OGD-PBMCs administration *in vivo.*

SSEA-3-positive cell recruitment in the ischemic cerebral cortices 10 days after ischemia (administered after 3 days). Although lower magnification images qualitatively revealed the overall status of the tissue, the differences among the groups were not quantitative. Scale bar, 50 μm.

Supplementary Figure 7

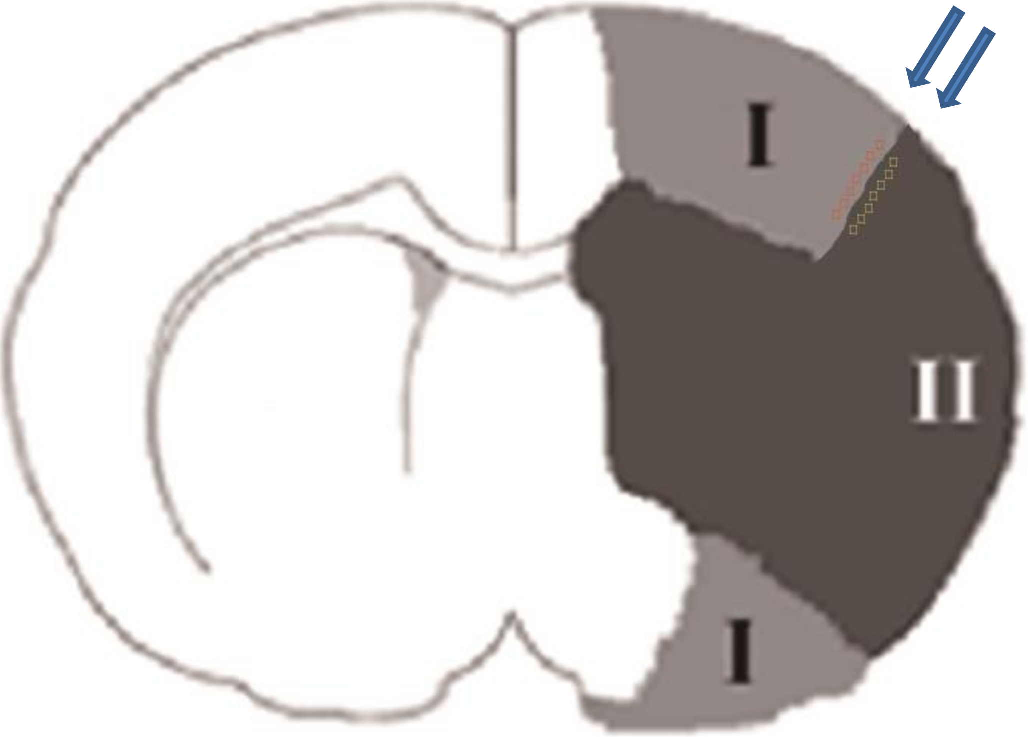

Supplementary Figure 7. Immunohistochemical analyses in the ischemic core and penumbra.

Cortical tissues belonging to the ischemic penumbra (region I) and core (region II) were evaluated. The rat focal cerebral ischemia model provides an area of the ischemic core and penumbra determined by the presence of MAP2 with a high degree of reproducibility. To perform quantitative analyses of brain tissue structures, tissue sections were immunostained with antibodies against CD31 (a marker of endothelial cells and angiogenesis), SMI31 (markers of neuronal axons), and VEGF, and SSEA-3 and TGF-β-positive cells were counted as described previously [9,35,36]. Briefly, seven randomly chosen non-overlapping high-power fields (630×) at the level of the anterior commissure of the sham-operated or ischemic cortex in the MCA territory were examined (yellow boxes in the ischemic core, red boxes in the ischemic penumbra) (arrow).

References

9. Kanazawa, M. et al. Microglia preconditioned by oxygen-glucose deprivation promote functional recovery in ischemic rats. *Sci. Rep*. **7**, 42582 (2017).

35. Katchanov, J. et al. Mild cerebral ischemia induces loss of cyclin-dependent kinase inhibitors and activation of cell cycle machinery before delayed neuronal cell death. *J. Neurosci.* **21,** 5045–5053 (2001).

36. Kanazawa, M. et al. Biochemical and histopathological alterations in TAR DNA binding protein-43 after acute ischemic stroke in rats. *J. Neurochem.* **116,** 957–965 (2011).

.

Supplementary Figure 8

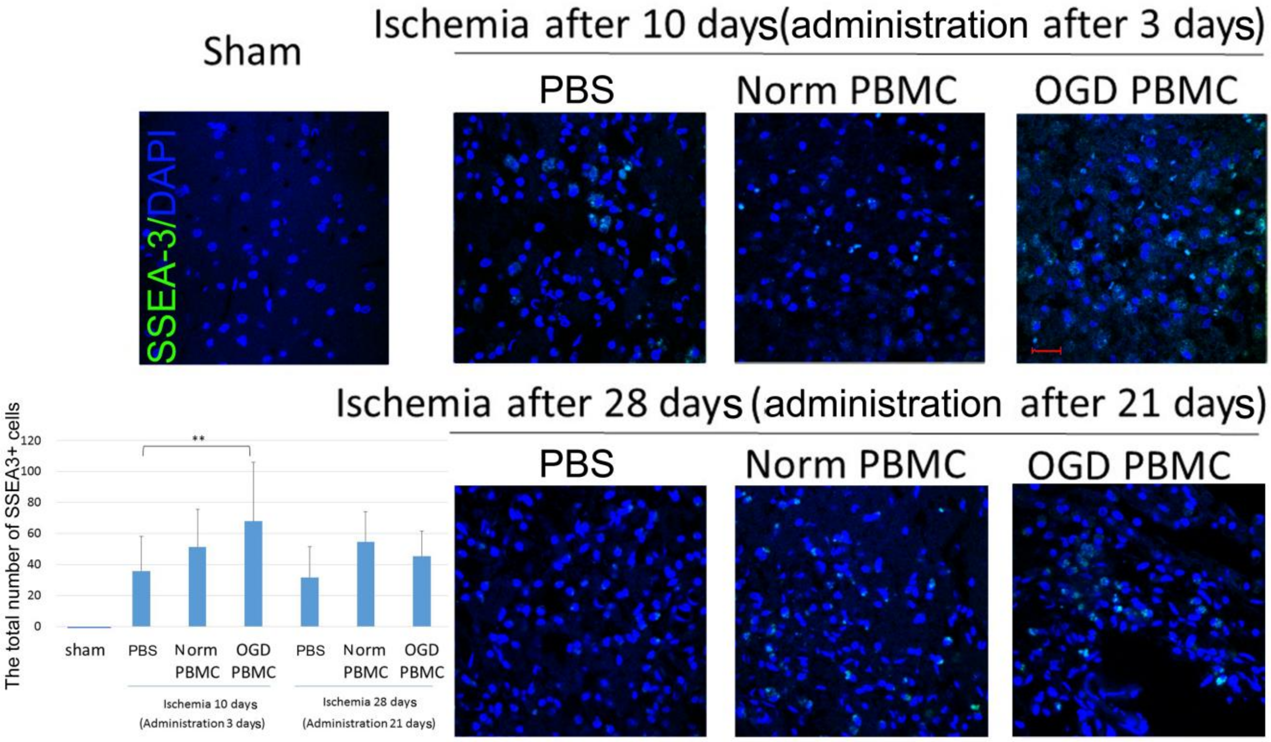

Supplementary Figure 8. Increasing number of SSEA-3-positive cells after OGD-PBMCs administration *in vivo.*

SSEA-3-positive cell recruitment in the sham-operated and ischemic cerebral cortices at 10 days (administration after 3 days) and 28 days after ischemia (administration after 21 days). A bar graph represents the total number of SSEA-3-positive cells. Statistical analyses were performed by one-way ANOVA. Scale bar, 20 μm. **P < 0.01 (N = 28).

Supplementary Figure 9

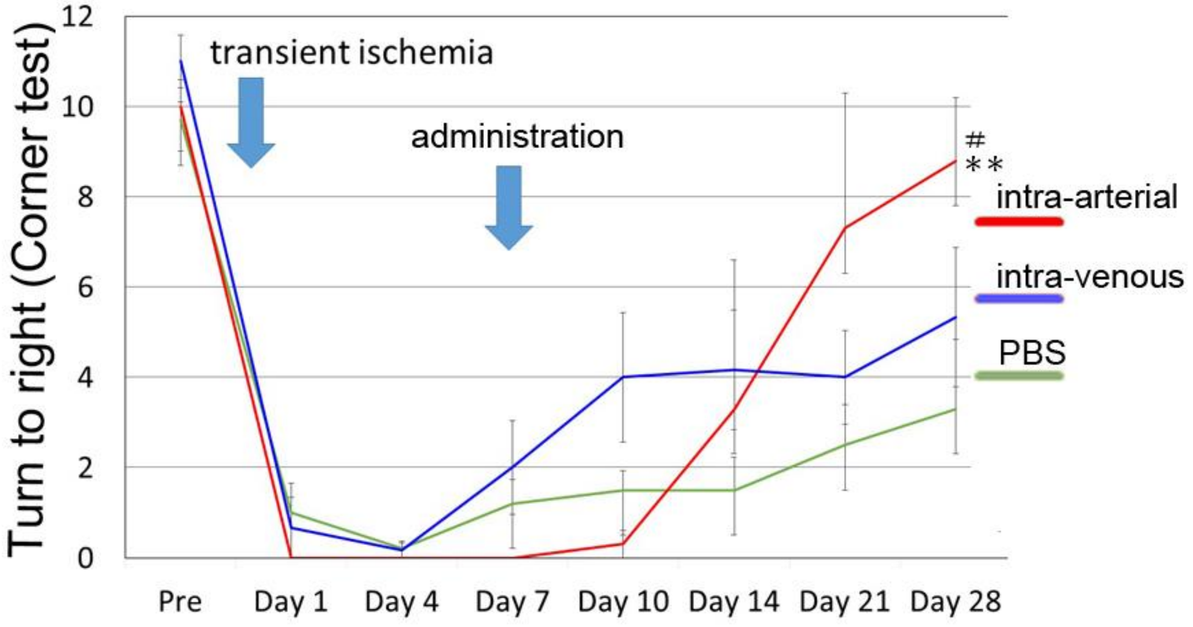

Supplementary Figure 9. Comparison of neurological outcomes after administration of OGD-PBMCs between via intra-arterial route and via intra-venous route.

Better functional recovery on the corner test (performed 20 times) was observed in the intra-arterially administrated OGD-PBMCs group (intra-arterial group, N = 8) compared with the vehicle intra-arterially administrated control group (PBS control group, N = 6) and intra-venously administrated OGD-PBMCs group (intra-venous group, N = 6) at 28 days after cerebral ischemia. Statistical analyses were performed by one-way ANOVA.; $^{\#}P = 0.06$ vs intra-venous group, $^{**}P < 0.01$ vs PBS control group ($N \geq 6$ per group). No functional difference was observed between intra-venous group and PBS control group ($P = 0.38$).
